# Significance of potential inflammatory foci in the oral cavity during dental examination in patients qualified for cardiac surgery – present study

**DOI:** 10.1186/1749-8090-8-S1-O107

**Published:** 2013-09-11

**Authors:** J Konstanty-Kalandyk, A Kalandyk-Konstanty, B Kapelak, R Drwila, J Zarzecka, J Piatek, J Sadowski

**Affiliations:** 1 Department of Cardiovacular Surgery and Transplantology, John Paul II Hospital, Cracow, Poland; 2 Department of Conservative Dentistry and Endodontics, Collegium Medicum Jagiellonian University, Cracow, Poland

## Background

Recent guidelines for treatment and prevention (AHA) infective endocarditis underline the importance of proper oral hygiene and the presence of the primary foci of inflammation in patients scheduled for surgery. The aim of the study is to assess the state of oral health and treatment needs of patients referred for cardiac surgery, development of a standard dental examination and comparative analysis of the type and frequency of bacterial complications between the group of patients without and after oral sanitation in the postoperative period.

## Methods

The study included 669 patients operated on in the Department of Cardiovascular Surgery and Transplantology. Patients were divided into two groups: study group - 243 patients, who will have done screening for the presence of inflammatory foci, based on prepared dental examination protocol and a control group - 426 patients.

## Results

In the entire study group 50% had a current certificate from the dentist, 33% brushes his teeth once a day or less and 87% goes to the dentist at least once a year.

Among the patients included in the study, only 10% had not the potential inflammatory foci and did not require any intervention dentistry. The average value of EuroSCORE in the study group was 2.66% and the median age of patients was 60 years. 25% of patients were diabetic. The postoperative infectious complications in the study group occurred in 4 patients (1 patient (0.4%) - an infection of the sternum, and 3 patients (1.2%) - wound infection). 5 (2%) patients required re-suture the sternum due to infection after leaving the hospital. In patients with diabetes, infection occurred in 1.6% in the postoperative period and at 4.9% thereafter.

## Conclusions

Oral hygiene in Poland, in patients undergoing cardiac surgery is bad. Proper assessment of potential inflammatory foci in the mouth will help to reduce the risk of infections in the postoperative period, which is particularly important in patients with diabetes.

